# Transcriptional and physiological data revealed cold tolerance in a photo-thermo sensitive genic male sterile line Yu17S

**DOI:** 10.1186/s12870-022-03437-8

**Published:** 2022-01-21

**Authors:** Xiaoxue Pan, Ling Guan, Kairong Lei, Jingyong Li, Xianwei Zhang

**Affiliations:** 1 Biotechnology Research Institute, Chongqing Academy of Agricultural Sciences/Chongqing Key Laboratory of Adversity Agriculture, Chongqing, 401329 China; 2 Chongqing Rationing Rice Research Center, Chongqing Academy of Agricultural Sciences, Chongqing, 402160 China

**Keywords:** PTGMS lines, Chilling stress, Transcriptional profiles, Carotenoid biosynthesis, Abscisic acid biosynthesis

## Abstract

**Background:**

Rice is highly sensitive to chilling stress during the seedling stage. However, the adaptable photo-thermo sensitive genic male sterile (PTGMS) rice line, Yu17S, exhibits tolerance to low temperatures. Currently, the molecular characteristics of Yu17S are unclear.

**Results:**

To evaluate the molecular mechanisms behind cold responses in rice seedlings, a comparative transcriptome analysis was performed in Yu17S during seedling development under normal temperature and low temperature conditions. In total, 9317 differentially expressed genes were detected. Gene ontology and pathway analyses revealed that these genes were involved mostly in photosynthesis, carotenoid biosynthesis, carbohydrate metabolism and plant hormone signal transduction. An integrated analysis of specific pathways combined with physiological data indicated that rice seedlings improved the performance of photosystem II when exposed to cold conditions. Genes involved in starch degradation and sucrose metabolism were activated in rice plants exposed to cold stress treatments, which was accompanied by the accumulation of soluble sugar, trehalose, raffinose and galactinol. Furthermore, chilling stress induced the expression of phytoene desaturase, 15-cis-ζ-carotene isomerase, ζ-carotene desaturase, carotenoid isomerase and β-carotene hydroxylase; this was coupled with the activation of carotenoid synthase activity and increases in abscisic acid (ABA) levels in rice seedlings.

**Conclusions:**

Our results suggest that Yu17S exhibited better tolerance to cold stress with the activation of carotenoid synthase activity and increasing of ABA levels, and as well as the expression of photosynthesis-related genes under cold condition in rice seedlings.

**Supplementary Information:**

The online version contains supplementary material available at 10.1186/s12870-022-03437-8.

## Introduction

Rice (*Oryza sativa* L.) is one of the most important food crops in the world and feeds more than 50% of the world’s population [1, 2]. As a tropical plant, rice is more sensitive to cold stress than other cereal crops such as wheat (*Triticum aestivum*. L) and barley (*Hordeum vulgare.* L) [3]. Rice seedling establishment is negatively affected by exposure to low temperatures. For example, during the direct sowing of early rice and in double-cropping rice regions, rice seedlings are often affected by ‘late spring coldness’ weather in March and April, which causes yellow leaves, slow seedling growth, stunting, withering and reduced tiller production, all of which ultimately affect rice production [4]. Asian rice consists of two subspecies, *Indica* and *Japonica*, which differ in their tolerances to low temperature stress [5–7]. *Indica* rice cultivars, which are distributed mainly in tropical and subtropical regions, are more sensitive to cold stress compared with *Japonica* cultivars. Intriguingly, farmers in tropical and subtropical areas of China prefer to grow *Indica* rice cultivars [8]. As a consequence, high-yield *Indica* rice cultivars possessing cold stress tolerance are highly desirable in these regions.

Upon sensing environmental stress, plants sacrifice growth and activate protective responses to the stress [9]. For example, environmental stressors such as drought, salt and low temperature induce endogenous abscisic acid (ABA) accumulation [10–12], and the application of exogenous ABA improves the cold resistance of plants [13–15]. To date, transcriptomic analyses in various plant species have identified several stress-responsive metabolites [4, 16–18], such as chlorophyll, osmoprotectants and plant endogenous hormones [19–25]. ABA is produced via the carotenoid pathway in plastids. Mutations in phytoene synthase (*OsPSY*), phytoene desaturase (*OsPDS*), 15-cis-ζ-carotene isomerase (*OsZ-ISO/MIT1*), ζ-carotene desaturase (*OsZDS*), carotenoid isomerase (*OsCRTISO*) and β-carotene hydroxylase (*OsBCH*/*DSM2*) block the biosynthesis of carotenoid precursors, thereby reducing ABA accumulation [26–30]. The initial step in ABA biosynthesis is the hydroxylation of all-*trans*-β-carotene into zeaxanthin, which is then converted to all-*trans*-violaxanthin by zeaxanthin epoxidase encoded by *ABA DEFICIENT1* (*ABA1*). All-*trans*-violaxanthin is subsequently converted to all-*trans*-neoxanthin by *ABA4* in *Arabidopsis* [31]. Violaxanthin and neoxanthin further undergo oxidative cleavage to form xanthoxin, which is catalyzed by one or more 9-*cis*-epoxycarotenoid dioxygenases (NCEDs); this is the rate-limiting step in de novo ABA biosynthesis [32, 33]. ABA induces the expression of stress-related genes, promotes stomatal closure and increases the production of specific carbohydrates, including sucrose, hexose, raffinose, glucose, fructose and trehalose [34].

In recent years, microarrays and high-throughput RNA sequencing (RNA-Seq) have been applied to examine the transcriptomes of rice exposed to low temperature stress [4, 35–38]. Cold responsive genes and pathways vary among cold tolerant rice cultivars, although there are some common responses to low temperatures. The photo-thermo sensitive genic male-sterility (PTGMS) rice line is an important component of the two-line hybrid rice system. Many PTGMS rice line studies have focused on the mechanisms underlying the low critical sterility-inducing temperature (CSIT) of the PTGMS line. However, little information is available regarding the molecular responses of PTGMS rice line seedlings to chilling stress.

A novel PTGMS line, Yu17S (spp. *indica*), possesses high tolerance to chilling stress and CSIT < 23 °C [39]. In this study, Yu17S was crossed with two *indica* type restorer lines, Huazhan and Minghui63. All F_1_ hybrids exhibited cold tolerance, moderate plant types, good eating quality, and the potential for high yields and resistance to rice blast. To better understand the mechanisms driving Yu17S cold tolerance during the seedling stage, we investigated the transcriptomic changes in Yu17S seedlings subjected to cold stress using RNA-Seq. Based on our physiological, chemical and transcriptomic data, we found that genes related to carotenoid and ABA biosynthesis, starch degradation, sucrose metabolism and photosynthesis were differentially expressed in cold-stressed Yu17S.

## Methods

### Plant material and low temperature treatment

The cold susceptible cultivar, MH63 (Minghui63, an *indica* restorer line) were collected from Sanming Academy of Agricultural Sciences, Sanming, Fuiang, China, and the cold-tolerant PTGMS line Yu17S (an *indica* cultivar bred by the Chongqing Academy of Agricultural Sciences) was cultivated in Yongchuan (29°23′ N, 105°53′ E) and Lingshui (18°30′ N, 110°01′ E) of China. The seeds of each genotype were grown hydroponically in Yoshida’s culture solution [40] in a growth chamber (MMM, Neuss, Germany) at a constant 26 °C day/night temperature under a 14 h photoperiod (220 μmol photons/m^2^/s). For the cold treatment, 3-leaf-stage plants were exposed to 4 °C and 24 h illumination. Seedling samples were collected at 0, 2, 6, 12, 24 and 48 h time points during cold stress. After 48 h growth at 4 °C, the plants were transferred to 26 °C for 24 h to assess recovery. Samples were frozen in liquid nitrogen and stored at − 80 °C.

### RNA sequencing

A total of 21 Yu17S seedling samples (seven time points, three biological replicates at each time point) were sequenced, and paired-end sequencing was performed by the Novogene Biotechnology Corporation (Beijing, China) using the Illumina Novaseq™ 6000 platform. For each sample, 40–56 million raw reads were obtained (Additional file 1: Table S1). Prior to assembly, low quality reads were removed using FastQC (v0.11.5) [41], and then the clean reads were aligned to the Ensembl reference genome (ftp://ftp.ensemblgenomes.org/pub/plants/release-49/fasta/oryza_sativa/dna/) using HISAT2 software (v2.1.0) [42].

The transcriptomes of all samples were merged to reconstruct a comprehensive transcriptome using Perl scripts. From the reconstructed transcriptome, the mRNA expression levels were calculated by determining the fragments per kilobase of exon per million fragments mapped (FPKM) for each transcript using the R package Rsubread v1.22.2 [43]. Genes were deemed to be differentially expressed if transcripts exhibited a log2 fold-change > 1 or < − 1 and *P* < 0.05; this was calculated using the R package edgeR [44]. Furthermore, only differentially expressed genes (DEGs) with FPKM values ≥10 were analyzed (Additional file 2: Table S2). The molecular function and biological process gene ontology (GO) terms associated with DEGs were determined using agriGO (http://bioinfo.cau.edu.cn/agriGO/index.php), with the *P*-value parameter set to < 0.05. Enriched pathways associated with the DEGs were analyzed using the R package ClusterProfiler.

### Quantitative real-time PCR analysis

Quantitative real-time PCR (qRT-PCR) was performed to confirm the expression profiles of cold-responsive genes identified in RNA-seq. Total RNA was extracted from samples using TRIzol reagent (Invitrogen, Carlsbad, CA, US) and purified using RNase-Free DNase I (Roche, Basel, Switzerland). RNA was quantified using the NanoDrop ND-1000 (Thermo Scientific, Waltham, MA, USA). The first-strand cDNA was generated from 1 μg total RNA using M-MLV reverse transcriptase (Promega, Madison, WI, US), and qRT-PCR was performed on the BIO-RADCFX96 Real Time System (Bio-Rad Laboratories, Hercules, CA, US) using a SYBR green PCR kit (DRR041A, TaKaRa, Shiga, Japan). Primer sequences are listed in Additional file 3: Table S3. The relative gene expression levels were calculated using the 2^ΔΔCT^ method with normalization to the level of *ACTIN 1* (*OsAct1*; LOC_Os05g36290) [45, 46]. Three independent experiments were performed for the qRT-PCR analysis.

### Physiological and biochemical assays

Chlorophyll fluorescence was measured using the IMAGING-PAM (WALZ, Germany), and maximum photochemical efficiency was measured using the default parameters according to the following equation: Fv/Fm = (Fm − F0)/Fm. Plants were placed in the dark 20 min before measurements and then 25 individual seedlings were analyzed.

Endogenous ABA was measured using the procedure as described previously with minor revision [47]. In brief, the 100 mg fresh leaves samples were collected and ground in tissue grinder (Jingxin, China). Frozen powder was added with 750 μl buffer (methanol: ddH_2_O: acetic acid (80:19:1) (V: V: V)) together with 7.5 ng D6-ABA (internal standard) (Sigma, USA) and was extracted overnight at 4 °C and then centrifuged for 10 min at 13000 rpm at 4 °C. The supernatant was then transferred to a new tube and evaporated under nitrogen gas. The residue was dissolved with 200 μl 30% methanol, and ABA were determined by high performance liquid chromatography mass spectrometry (HPLC–MS) system with an Agilent 1290 system (Agilent Technologies, USA) coupled with a 6500 QTRAP system(AB SCIEX, USA) [17].

For the determination of raffinose and galactinol, 10 g of fresh leaves were ground with liquid nitrogen and extracted with 10 ml distilled water for 30 min at 80 °C. After centrifugation at 5000 g for 10 min, the supernatant was added to 40 mL ethanol, mixed, and left overnight at 4 °C. Subsequently, centrifugation at 5000 g for 10 min, the supernatant was filtered three times with a 0.22 μm filter membrane. The extract was dried and dissolved in 1 ml distilled water, and the product were determined by HPLC (Waters, USA) with evaporative light scattering detector (ELSD, Alltech 3300, USA) [17, 48, 49].

Analysis kits from Nanjing Jiancheng Bioengineering Institute (Nanjing, China) were used to measure the contents of chlorophyll, carotenoids, total soluble sugar and trehalose. Three independent experiments were used in the determination of ABA, raffinose, galactinol and physiological tests of leaves in control and chilling treated seedlings.

## Results

### Yu17S is more tolerant to cold stress than MH63

**Fig. 1:**
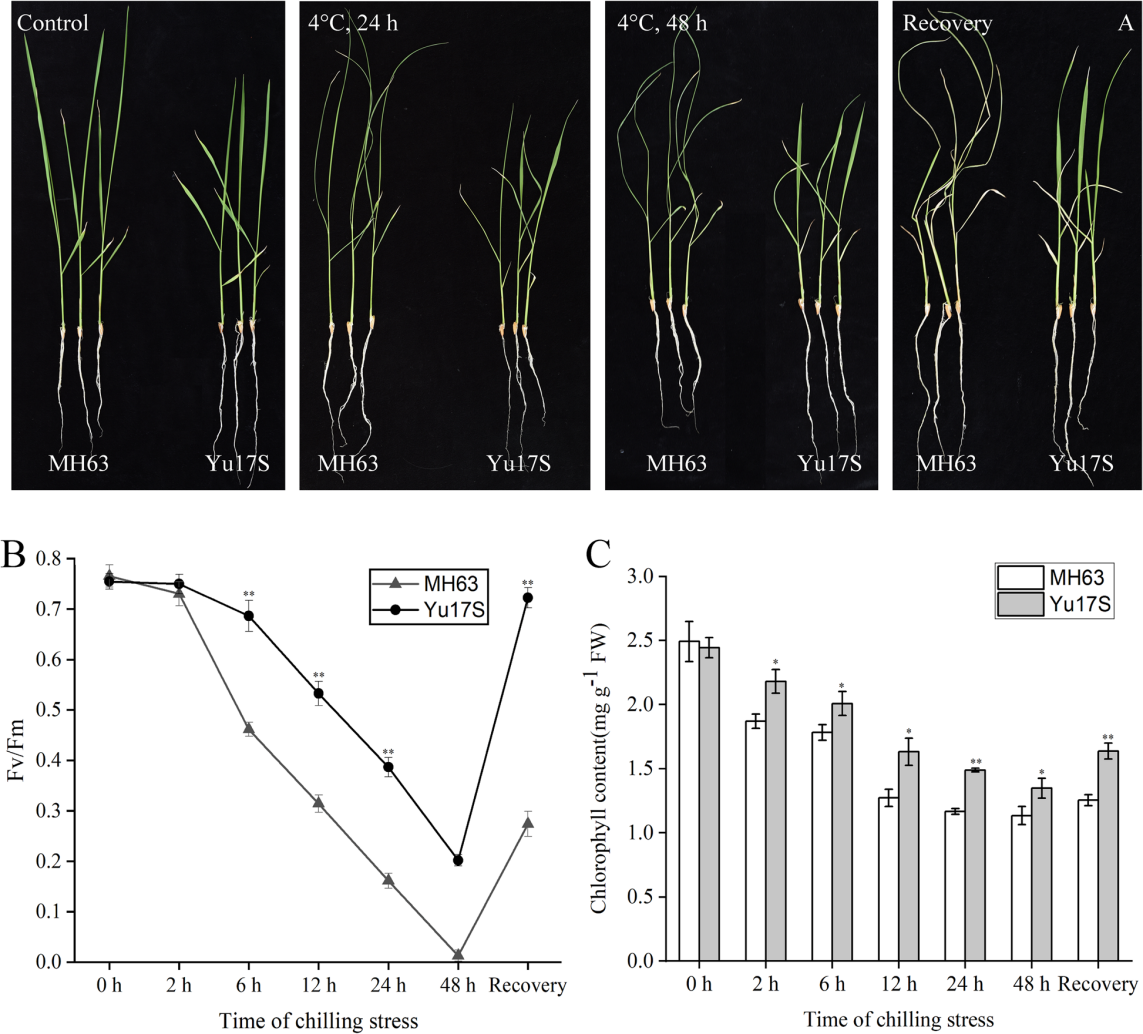
Phenotypes of two rice genotypes under chilling stress and subsequent recovery. **A** Comparison of seedlings of Yu17S and MH63 in control, treated at 4 °C for 12 h, 48 h and recovery for 24 h after 48 h treatment. **B** Determination of photochemical efficiency of PS II system in Yu17S and MH63 under cold stress (2–48 h) and recovery for 24 h after 48 h treatment. **C** Chlorophyll content in Yu17S and MH63 under cold stress (2–48 h) and recovery for 24 h after 48 h treatment. FW, fresh weight; Error bars indicate the SD for three independent replicates. * and ** indicate a significant difference between MH63 and Yu17S at *P* < 0.05 and *P* < 0.01 levels, respectively (two tailed T-test)

Following exposure to low temperatures, the survival rates of Yu17S and MH63 were significantly different, with Yu17S exhibiting better cold tolerance and ability to recover compared with MH63 (Fig. 1). MH63 seedlings exhibited obvious signs of death and wilting, while only a few Yu17S seedlings died (Fig. 1A). There was no significant difference in the Fv/Fm ratio between Yu17S and MH63 before cold exposure (0.770 vs. 0.765). However, we found that the Fv/Fm was significantly lower in MH63 than in Yu17S after 48 h cold exposure and recovery; the Fv/Fm after 48 h cold exposure and recovery were 0.253 and 0.723 for Yu17S versus almost zero and 0.274 for MH63, respectively (Fig. 1B). These results suggest that Yu17S has higher cold tolerance compared with MH63.

### Identification of DEGs under cold stress

RNA-seq of 21 samples generated 1,007,527,684 raw reads, with an average of 47,977,508 reads per sample (Additional file 1: Table S1). The GC contents of the transcriptomes ranged from 50.32 to 53.04%, with an average of 51.89%. The Q30 values of the 21 samples were 92.11–95.88%, with an average of 94.71%. The high-quality reads of each sample were successfully aligned to the cv. Nipponbare (*Oryza sativa* L. *subsp. japonica*) reference genome, with a range of 87.20–93.18%.

**Fig. 2:**
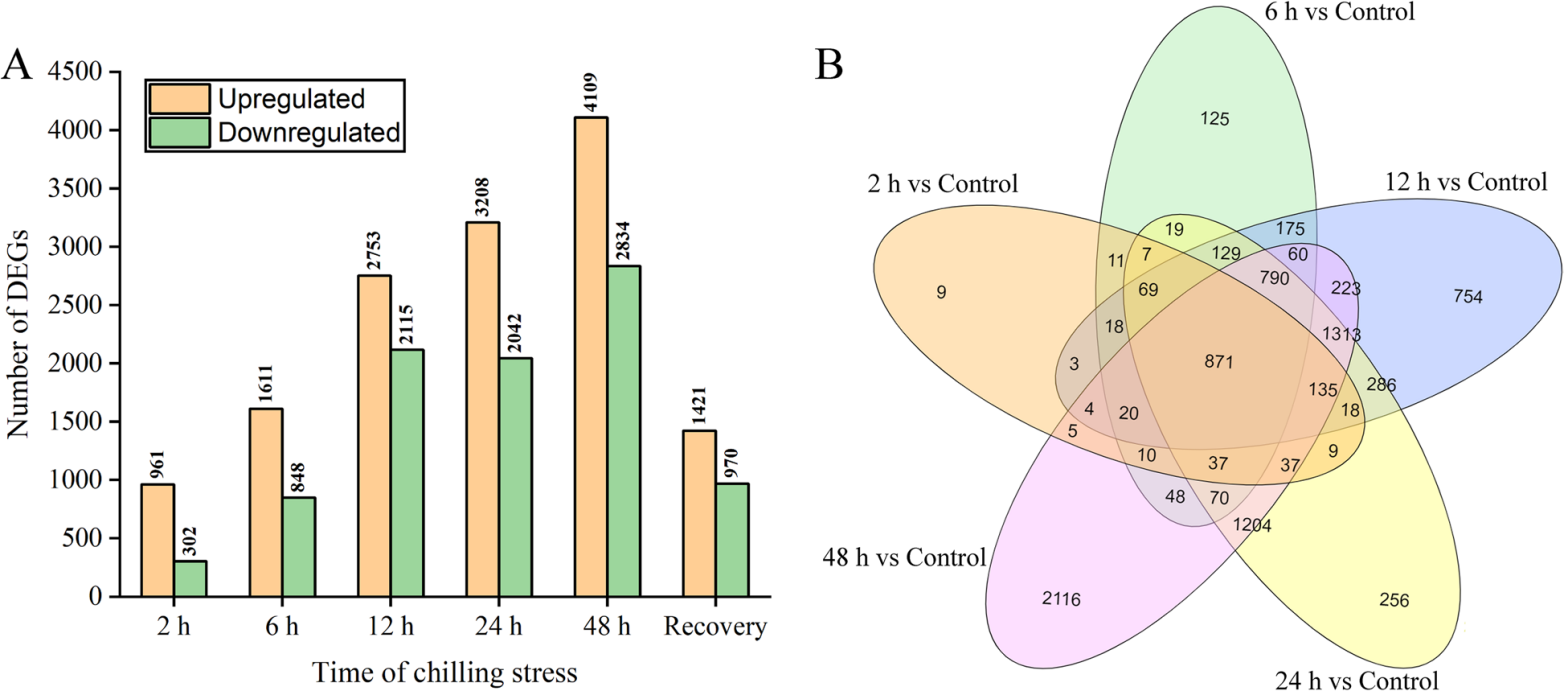
Transcriptomic overview of a time course of cold stress on Yu17S seedlings. **A** Total number of transcripts that were significantly up- or downregulated in response to cold stress. **B** Venn diagram illustrating the number of genes up- and downregulated under cold stress (2–48 h)

A total of 9317 DEGs were identified among the control, low temperature and recovery conditions. Among them, 8831 DEGs were identified in at least one cold condition, and 2391 DEGs were identified in the recovery condition compared with the control (Additional file 2: Table S2). In total, 1263 (961 up- and 302 downregulated), 2459 (1611 up- and 848 downregulated), 4868 (2753 up- and 2115 downregulated), 5250 (3208 up- and 2042 downregulated), 6943 (4109 up- and 2834 downregulated) and 2391 (1421 up- and 970 downregulated) DEGs were identified following 2, 6, 12, 24 and 48 h under cold conditions and after the 24 h recovery period, respectively (Fig. 2A, Additional file 2: Table S2). Of 8831 DEGs, 871 were differentially expressed at all five time points, and 9, 125, 754, 256 and 2116 were specifically affected at 2, 6, 12, 24 and 48 h of cold exposure, respectively (Fig. 2B). GO terms associated with transcription regulation (GO: 0006350) such as transcription factors (103, 11.83%) were enriched among 871 DEGs. The transcription factors could be divided into 37 different common families, of which 97.08% were activated (Additional file 4: Table S4). Of these families, AP2-EREBP (10), bHLH (10) and NAC (10) families were the largest, followed by MYB-related (8), TIFY (8), WRKY (7) and HB (6) families.

**Fig. 3:**
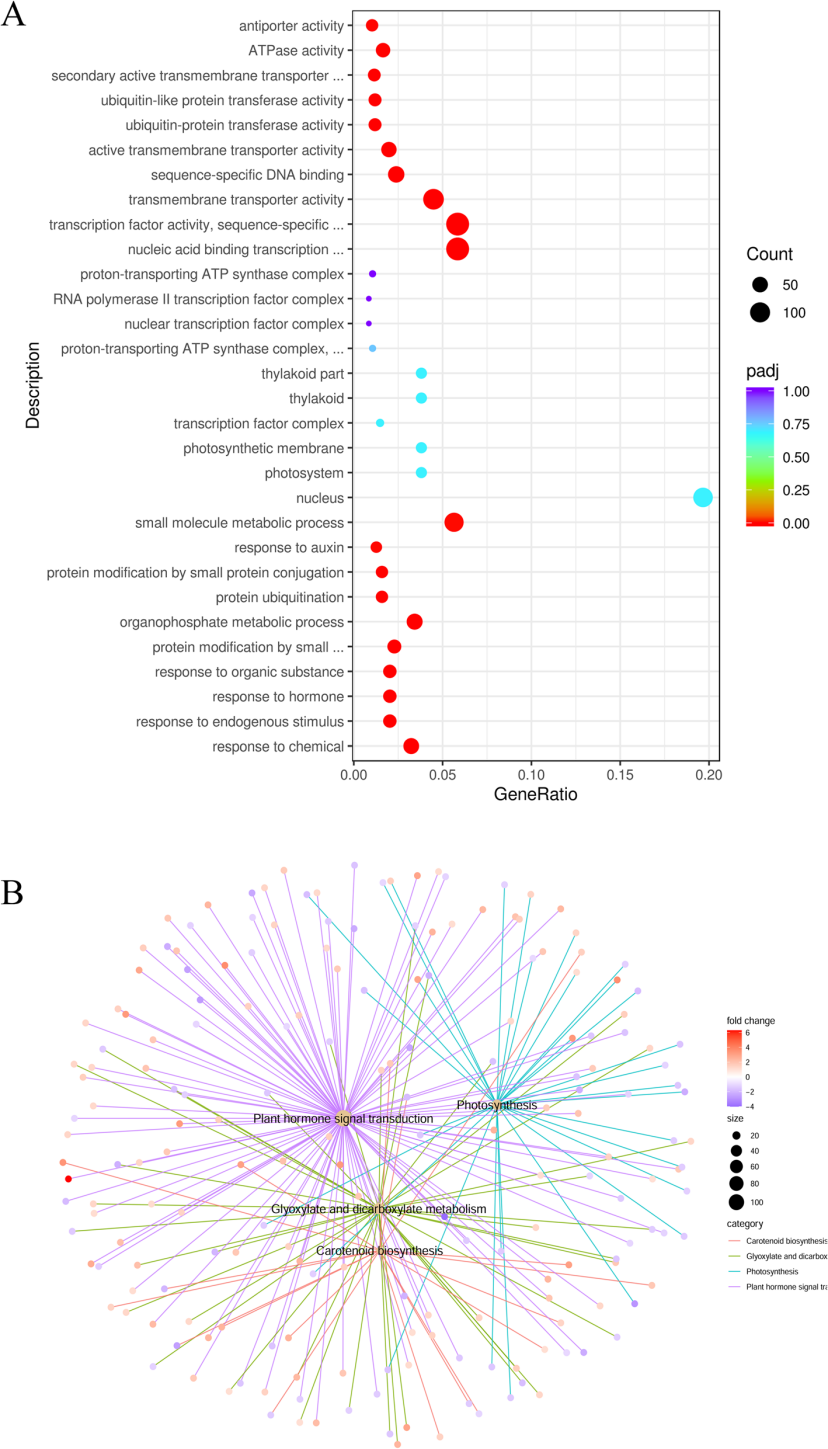
Functional annotations of transcriptome. **A** GO enrichment analysis of upregulated DEGs in Yu17S under cold stress (2–48 h). **B** KEGG enrichment analyses of all DEGs in Yu17S under cold stress (2–48 h) and recovery for 24 h after 48 h treatment

The 9317 DEGs were classified into 122 functional groups by the GO analysis, including 62 biological process and 60 molecular function categories (Additional file 5: Table S5). Under cold stress (2–48 h), many upregulated DEGs were associated with “response to hormone” (GO:0009725, 4.46E-09) and “protein modification” (GO:0070647, 3.40E-07) in the biological process GO category and with “transcription factor activity” (GO:0003700, 2.98E-23), “transmembrane transporter activity” (GO:0022857, 3.02E-10) and “sequence-specific DNA binding” (GO:0043565, 3.88E-10) in the molecular function GO category (Fig. 3A, Additional file 6: Table S6). Under the recovery condition, the main biological process associated with the downregulated DEGs was “photosynthesis” (GO: 0015979, 2.54E-12), while “chitin catabolic process” (GO: 0006032, 2.05E-05) was the most enriched term among the upregulated DEGs (Additional file 7: Table S7). Moreover, the DEGs were assigned to 115 pathways, with plant hormone signal transduction (osa04075), photosynthesis-antenna proteins (osa00196) and carotenoid biosynthesis (osa00906) playing vital roles in the seedling response to cold stress treatments. The carbon metabolism pathway (ko01200) was also enriched among the DEGs (Fig. 3B, Additional file 8: Table S8).

### Expression level of genes involved in the light reactions of photosynthesis for cold stress in Yu17S seedlings

**Fig. 4:**
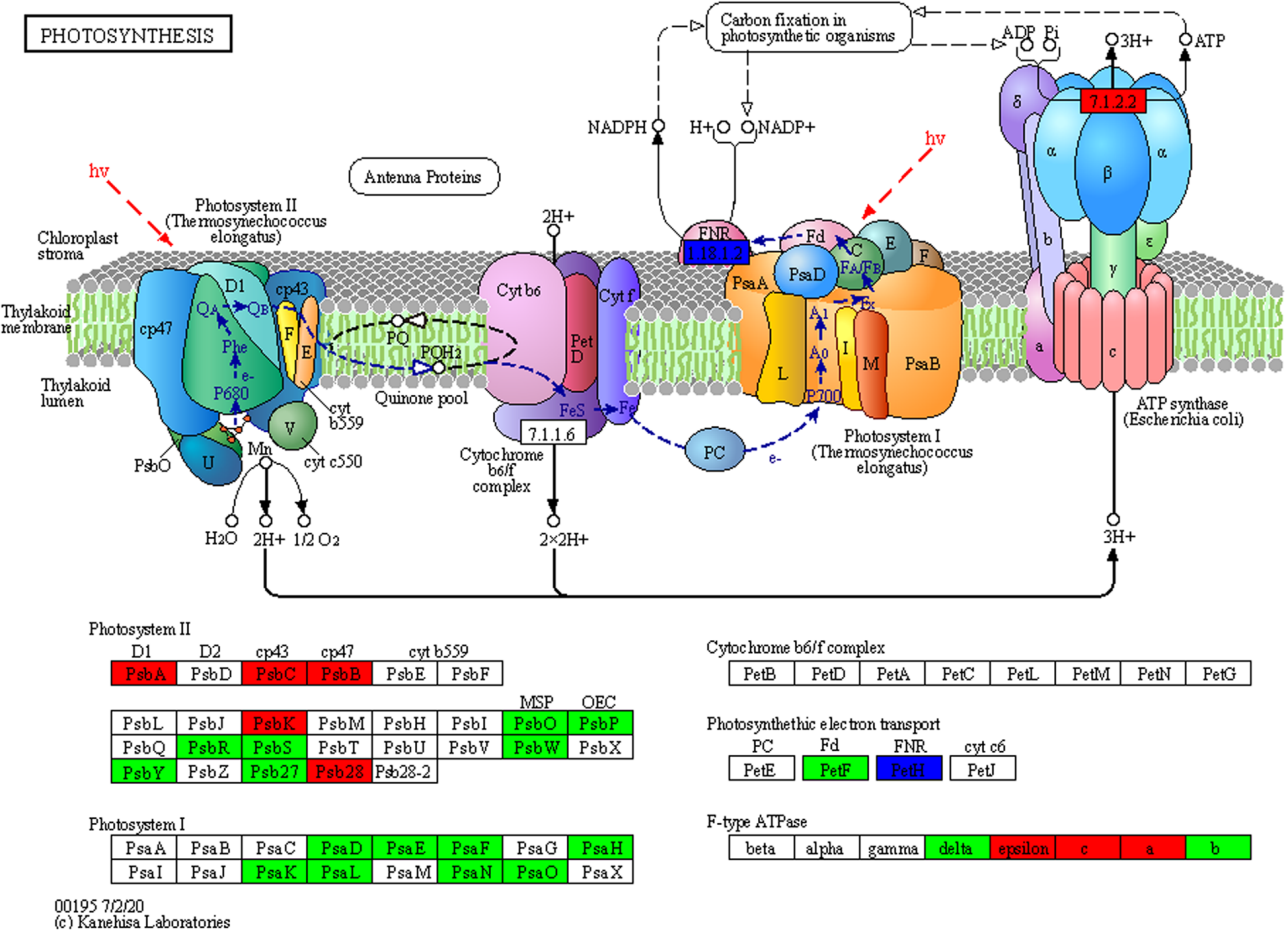
DEG-enrichment of the plant photosynthesis pathway by KEGG annotation. The key regulatory components in photosynthesis pathways are presented as their names (red, upregulated; green, downregulated). Gene IDs and fold changes in transcript abundance are indicated in Additional file 9 Table S9

Transcriptome analysis indicated that the photosynthetic processes of rice seedlings were affected by cold stress exposure, and the transcript level of many light reactions of photosynthesis genes, such as photosystem I, photosystem II, ferredoxin-NADP reductase and adenosine triphosphate (ATP) synthase, were differentially regulated following cold stress (Fig. 4; Additional file 9: Table S9). Here, most genes encoding photosystem I (PSI) subunits and ferredoxin-NADP reductase were significantly downregulated in rice plants subjected to cold treatment (Fig. 4, Additional file 9: Table S9). However, genes encoding photosystem II (PSII) subunits (*psbA, psbB, psbC, psbK* and *psb28*) and ATP synthase proteins (*atpA*, *atpC* and *epsilon*) were dramatically upregulated under cold stress conditions (2–48 h) but also remained activated after 24 h growth under the recovery condition. These data were consistent with our pulse-amplitude-modulation measurements of maximum quantum efficiency of PSII photochemistry (Fv/Fm) (Fig. 1B), which indicated that Yu17S adapted to cold stress more rapidly than did MH63.

Chlorophyll has unique and essential functions in photosynthetic light harvesting and energy transduction [50]. In this study, we also found that the transcript levels of several chlorophyll A-B antenna proteins were significantly downregulated, but we detected three early light-induced proteins (ELIP) upregulated in Yu17S during cold stress (Additional file 9: Table S9). There was no significant difference in the total chlorophyll content between Yu17S and MH63 after 6 h cold exposure. However, the total chlorophyll content was significantly lower in MH63 than in Yu17S after 48 h cold exposure and recovery; the total chlorophyll contents after 48 h cold exposure and recovery were 14.2 and 39.1 mg g^− 1^ fresh weight for MH63 versus 25.8 and 50.2 mg g^− 1^ fresh weight for Yu17S, respectively (Fig. 1C), suggesting that the higher chlorophyll content serve the photosynthetic activity in Yu17S seedlings following cold stress.

### Expression level of genes involved in plant hormone signal transduction under Yu17S seedlings of cold stress

Both the GO and pathway analyses identified plant hormone biosynthesis and signal transduction (GO: 0007165, osa04075) as enriched pathways in rice seedlings under cold stress (Fig. 3). In total, 83 genes involved in plant hormone biosynthesis and 118 genes involved in plant hormone signal transduction exhibited altered expression in plants under cold conditions (Additional file 10: Table S10). The transcript levels of several gibberellic acid (GA) biosynthesis genes decreased following cold exposure, while those associated with gibberellic acid catabolism increased. The expression of genes involved in auxin, ethylene and jasmonic acid signaling was also activated under cold conditions. Moreover, genes associated with ABA signaling, including two ABA receptors of the PYRABACTIN RESISTANCE1 (PYR1)/PYR1-LIKE (PYL) family, two ABA-responsive element binding factors (ABFs), four protein phosphatase 2C (PP2C) genes and five serine/threonine-protein kinase SRK2 (SnRK2) genes, were upregulated during cold treatment (Additional file 10: Table S10). The above results indicated that plant hormones were participated in the response to cold stress in Yu17S.

### Both ABA and carotenoids level were enhanced in Yu17S seedlings during cold stress

**Fig. 5:**
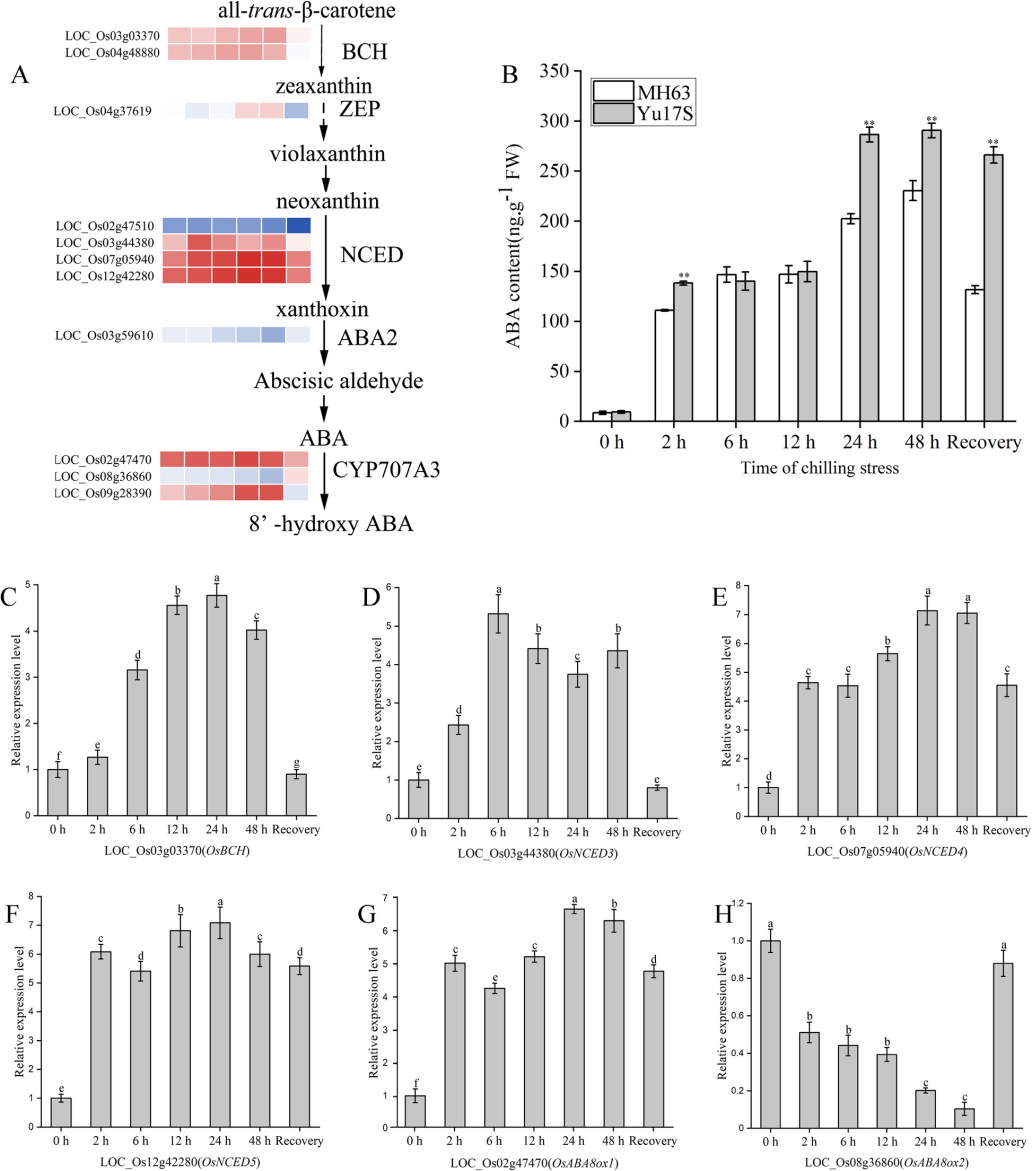
Overview of putative ABA metabolism pathways involved in rice and expression profiles of genes involved in this pathway. **A** The schematic of ABA metabolism pathway was shown in brief. **B** Detection of endogenous ABA contents in Yu17S and MH63 under cold stress (2–48 h) and recovery for 24 h after 48 h treatment. **C**-**H** Relative expression of genes involved in ABA metabolism pathway. Error bars indicate the SD for three independent replicates. Different letters indicate significant differences among treatments (P < 0.05, ANOVA). * and ** indicate a significant difference between MH63 and Yu17S at P < 0.05 and P < 0.01 levels, respectively (two tailed T-test). FW, fresh weight; BCH, β-carotene hydroxylase; ZEP, zeaxanthin epoxidase; ABA2, short-chain alcohol dehydrogenase; NCED, 9-*cis*-epoxycarotenoid dioxygenase; ABA, abscisic acid; YP707A3, Cytochrome P450, family 707, subfamily A, polypeptide 3

ABA plays important roles in the cold stress have been studied intensively. Here, of NCEDs are the rate-limiting enzymes in ABA biosynthesis, while cytochrome P450 707A family member (CYP707A) (ABA 8′-hydroxylase: ABA8ox) and ABA glucosyltransferase are involved in ABA catabolism [10, 31]. Of the genes encoding NCED family members, *OsNCED3* (LOC_Os03g44380), *OsNCED4* (LOC_Os07g05940) and *OsNCED5* (LOC_Os12g42280) were significantly upregulated under cold conditions. In addition, the transcript levels of *OsABA8ox1* (LOC_Os02g47470) and *OsABA8ox3* (LOC_Os09g28390) increased during cold treatment (2–48 h) and decreased slightly after the 24 h recovery period (Fig. 5A). qRT-PCR showed that the transcript levels of *OsBCH*, *OsNCED3*, *OsNCED4*, *OsNCED5* and *OsABA8ox1* were higher at 2, 6, 12, 24 and 48 h of cold stress compared with the control, but lower in *OsABA8ox3* (Fig. 5C–H). To confirm upregulation of the ABA biosynthetic pathway in Yu17S in response to cold stress, we quantified the endogenous ABA levels in the two cultivars (Yu17S and MH63) using HPLC–MS. At 0 h, there was no significant difference in the ABA level between Yu17S and MH63 (Fig. 5B). At 2 and 6 h of cold stress, the ABA content was slightly higher in Yu17S compared with MH63. The ABA levels in both strains increased sharply at 12 h and reached a maximum at 24 h under the recovery condition; however, the ABA content was lower in MH63 than in Yu17S. Compared with the 0 h control, the ABA contents of Yu17S and MH63 were approximately 10-fold and 4-fold higher after 24 h of recovery, respectively (Fig. 5B).

**Fig. 6:**
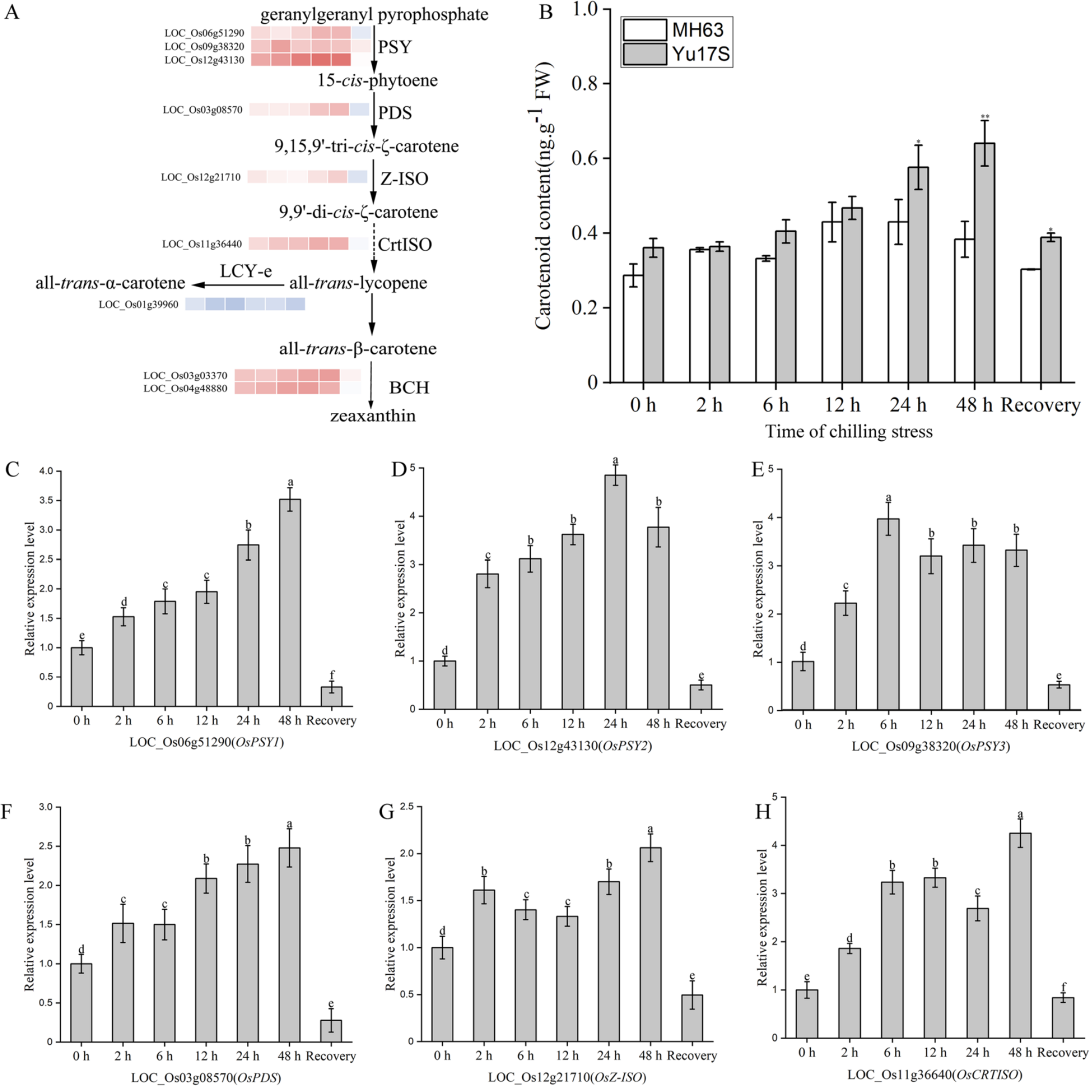
Overview of putative carotenoid biosynthesis pathways involved in rice and expression profiles of genes involved in this pathway. **A** The schematic of carotenoid biosynthesis pathway was shown in brief. **B** Carotenoid contents in Yu17S and MH63 under cold stress (2–48 h) and recovery for 24 h after 48 h treatment. **C**-**H** Relative expression of genes involved in carotenoid biosynthesis pathway. Error bars indicate the SD for three independent replicates. * and ** indicate a significant difference between MH63 and Yu17S at P < 0.05 and P < 0.01 levels, respectively (two tailed T-test). Different letters indicate significant differences among treatments (P < 0.05, ANOVA). FW, fresh weight; PSY, phytoene synthase; PDS, phytoene desaturase; Z-ISO, 15-*cis*-ζ-carotene isomerase; CRTISO, carotene isomerase; LCY-e, lycopene ε-cyclase; BCH, β-carotene hydroxylase

In plants, carotenoids are the precursor molecules of plant hormones such as ABA and strigolactone (BR) [30]. Our transcriptomic data revealed that several genes related to carotenoid biosynthesis were differentially expressed during cold exposure (Fig. 6A, Additional file 11: Table S11). For example, three *OsPSYs*, two *OsBCHs*, and *OsPDS*, *OsZ-ISO*, *OsCRTISO* were upregulated after cold stress treatment (Fig. 5A). The expression levels of LOC_Os06g51290, LOC_Os09g38320, LOC_Os12g43130, LOC_Os03g08570, LOC_Os11g36440 and LOC_Os12g21710 increased over the 2–48 h cold exposure period in Yu17S, but not in recovery condition (Fig. 6C-H), suggesting that the carotenoid biosynthesis pathway was activated by low temperature in Yu17S.

To confirm whether carotenoid content was also increased in the Yu17S response to low temperature, we measured the total carotenoid levels in MH63 and Yu17S seedling subjected to cold stress for 0, 2, 6, 12, 24 and 48 h and to the 24 h recovery condition following 48 h cold stress (Fig. 6B). The carotenoid concentrations in Yu17S and MH63 differed significantly during cold treatment and the recovery condition. The carotenoid level in Yu17S seedlings increased significantly after 2 h cold exposure, which corresponded to the upregulated expression of carotenoid biosynthesis genes (Fig. 6A, C-H). By contrast, the carotenoid levels did not change significantly in the MH63 seedlings in response to cold stress (Fig. 6B), suggesting that carotenoids serve important protective functions in Yu17S seedlings following cold stress.

### Expression changes of genes involved in osmotic adjustment for cold stress in Yu17S seedlings

**Fig. 7:**
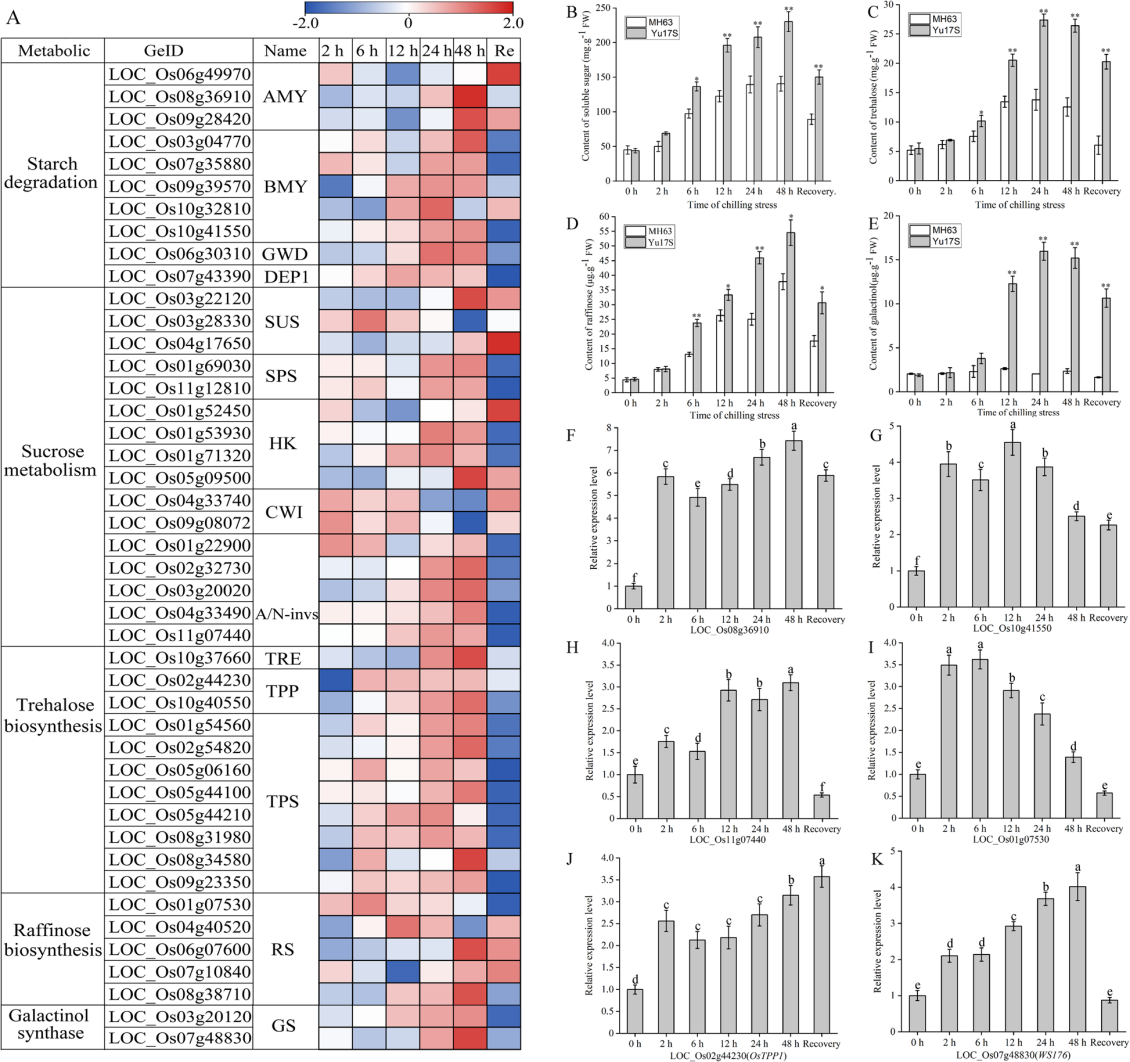
Carbohydrate metabolisms in Yu17S seedlings subject to chilling stress. **A** Heat maps illustrate transcript levels of representative cold responsive genes. The total soluble sugar (**B**), trehalose (**C**), raffinose (**D**) and galactinol (**E**) contents in the seedling of MH63 and Yu17S under cold stress (2–48 h) and recovery for 24 h after 48 h treatment. **F**-**K** Transcript levels of LOC_Os08g36910, LOC_Os10g41550, LOC_Os11g07440, LOC_Os01g07530, *OsTPP1* and *WSI76* in the seedling of Yu17S under cold stress (2–48 h) and recovery for 24 h after 48 h treatment. Error bars indicate the SD for three independent replicates. * and ** indicate a significant difference between MH63 and Yu17S at P < 0.05 and P < 0.01 levels, respectively (two tailed T-test). Different letters indicate significant differences among treatments (P < 0.05, ANOVA). FW, fresh weight; AMY, a-Amylase; BMY, b-amylase; GWD, glucan water dikinase; DEP1, disproportionating enzyme; SUS, Suc synthase; SPS, Suc-P synthase; HK, hexokinase; CWI, apoplastic invertase; A/N-Invs, alkaline/neutral invertase; TRE, trehalase; TPP, trehalose-6-phosphate phosphatase; TPS, trehalose-6-phosphate synthase; RS, raffinose synthase; GS, galactinol synthase

It has been well documented that the roles of sugar accumulation in plant response to cold stress [12, 22, 51]. The transcriptome analysis indicated that the carbohydrate metabolism processes of rice seedlings were affected by cold stress exposure (Fig. 7A). The soluble sugar level in Yu17S was significantly higher at 6–48 h of stress compared with 0 h, while that in MH63 was slightly higher at 12 h of cold stress compared with 0 h and then declined at 48 h (Fig. 7B). To examine this further, we screened cold responsive genes involved in starch degradation and sucrose metabolism (Fig. 7A, Additional file 12: Table S12). The transcript levels of most DEGs involved in starch degradation were elevated in rice plants after the onset of cold stress, especially α*-*amylase (LOC_Os08g36910, 7.25-fold increase after 2 h at 4 °C) and β-amylase (LOC_Os10g41550, 3.99-fold after 2 h at 4 °C). In the sucrose metabolism pathway, genes encoding sucrose synthase (SS), sucrose phosphate synthase (SPS) and alkaline/neutral invertases (NIN) were upregulated under cold conditions. The transcript levels of genes encoding trehalose-6-phosphate synthase (*OsTPS1)* and trehalose phosphate phosphatase (*OsTPP1)*, both crucial enzymes for synthesizing of trehalose, were increased significantly after cold treatment. The trehalose level in Yu17S was significantly higher at cold condition (12–48 h) and recovery condition compared with MH63 (Fig. 7C). We also examined the relationships between cold responsive gene expression and galactinol and raffinose biosynthesis. Four genes related to raffinose biosynthesis (LOC_Os01g07530, LOC_Os04g40520, LOC_Os06g07600 and LOC_Os08g38710) exhibited higher expression following cold exposure in Yu17S. The gene encoding galactinol synthase, *OsGolS1* (LOC_Os03g20120), *WSI76* (LOC_Os07g48830), were significantly upregulated under the cold conditions. Both genotypes exhibited an increase in raffinose levels after 2 h chilling stress compared with the controls, with higher raffinose contents detected in Yu17S than in MH63 (Fig. 7D). In contrast, galactinol contents was stable under both chilling and control conditions in MH63 and was increased in Yu17S after 6 h chilling stress compared with the control (Fig. 7E). Furthermore, high levels of LOC_Os08g36910, LOC_Os10g41550, LOC_Os11g07440, LOC_Os01g07530, *OsTPP1* and *WSI76* were detected by qRT-PCR in Yu17S during cold treatment (Fig. 7H-K; Additional file 12, Table S12); suggesting that these compounds was important in conferring osmotic adjustment in Yu17S seedlings under cold stress.

## Discussion

Cold tolerance is an important plant characteristic that enables adaptation to cold climates. In the present work, we performed RNA-seq analysis to identify the genes and pathways that participate in rice responses to cold stress at the seedling stage. Our findings indicated that important genes encoding enzymes involved in free sugar accumulation, photosynthesis, and carotenoid and ABA biosynthesis, are involved in cold resistance in rice seedlings.

Chlorophyll levels and Fv/Fm values are important markers of the tolerance to low temperature stress in rice [50]. Overexpression of stress-associated protein *OsiSAP8* in rice and tobacco (*Nicotiana tabacum* L.) significantly increases the plant chlorophyll content and improves the tolerance to low temperatures [19]. Overexpression of the ABA-stress- and ripening-induced gene *OsAsr1* significantly increased the Fv/Fm value in rice, which showed obvious growth advantages under low temperature conditions [20]. In this study, the Fv/Fm value and chlorophyll content decreased in the two genotypes under cold conditions over time; however, the decreases were slower in Yu17S than MH63 (Fig. 1B and C). Additionally, the transcriptional data indicated that many genes involved in photosynthesis, such as PSII components (*psbA, psbB, psbC, psbK and psb28*), ATP synthase subunits (*atpA, atpC and epsilon*) and early light-induced proteins, were significantly increased after cold exposure, while PSI and light-harvesting pathways were significantly lower at all cold exposure time points compared with the control (Fig. 4, Additional file 9: Table S9). Our data suggests that the higher chlorophyll and Fv/Fm values in Yu17S enabled the strain to maintain its photosynthetic properties, thus improving cold tolerance.

Carotenoid pigments can protect chlorophylls from adverse factors in plants [52]. Low temperature stress alters the accumulation of carotenoids in *Dunaliella salina* [53, 54], *Dunaliella bardawil* [55] and *Euglena gracilis* [56]. *OsPDS*, *OsZDS*, *OsCRTISO*, *OsBCH*/*DSM2* and *OsZ-ISO/MIT1* are key enzymes involved in the rice carotenoid biosynthetic pathway, and loss of function of these genes reduces the carotenoid content [26–30]. In the present study, the expression of *OsPSY1*, *OsPSY2*, *OsPSY3*, *OsPDS*, *OsCRTISO*, *OsBCH*/*DSM2* and *OsZ-ISO/MIT1* were significantly upregulated in rice seedlings subjected to cold exposure; as a result, carotenoids was accumulated in Yu17S during cold stress (Fig. 6, Additional file 11: Table S11). These results indicated that the carotenoid biosynthesis pathway was active during cold stress in Yu17S. In plants, carotenoids are the precursor molecules of ABA [30]. It has reported that carotenoid accumulation in response to osmotic, salt, dehydration and cold stress coincides with changes in ABA levels in plants [27, 30]. In the present study, the ABA level in Yu17S was significantly higher after cold treatment and during the recovery phase compared with the 0 h control (Fig. 5B). Moreover, the expression of *OsNCED3*, *OsNCED4* and *OsNCED5*, the rate-limiting enzyme of ABA biosynthesis, as well as 15 genes involved in ABA signal transduction (including *PYLs*, *ABF, PP2C* and *SnRK2*), was strongly induced by cold stress (Additional file 10: Table S10). Consistent with the higher ABA content, the transcript levels of two ABA-responsive genes, *OsLEA14/WSI18* and *OsLEA3* [12, 57, 58], two ABA responsive genes, were significantly higher in Yu17S (Additional file 2: Table S2). The induced expression of ABA biosynthesis genes and the increased endogenous ABA levels in our work indicated that the ABA-dependent transcription pathway may play critical roles in Yu17S at low temperatures. In our study, both carotenoids and ABA biosynthesis were enhanced during the cold acclimation process. However, whether carotenoids and ABA acts as the upstream regulatory factor during cold stress in Yu17S need to be determined in future studies.

The exogenous application of ABA increases the soluble sugar content of plants [59, 60]. Free sugars are stress-related metabolites that protect the plasma membrane from cold damage by regulating osmosis while providing energy for the synthesis of other organic substances [22, 25, 61]. The expression of key enzymes involved in the starch degradation pathway is activated during stress, increasing the production of maltose and other soluble sugars [62]. During low temperature exposure, the expression patterns of several genes related to starch degradation and sucrose metabolism increased dramatically, and these changes were correlated with the accumulation of soluble sugar [12, 51] (Fig. 7A). The transcript levels of LOC_Os08g36910 (encoding α-amylase), LOC_Os10g41550 (encoding β-amylase) and LOC_Os11g07440 (encoding analkaline/neutral invertase) were significantly higher under cold temperatures for up to 48 h (Fig. 7A), suggesting that they play an important role in starch degradation and sucrose metabolism in cold-stressed plants. Moreover, we observed increased expression levels of orthologs of genes involved in sucrose, trehalose, raffinose and galactinol synthesis, which supports the increased sucrose, galactinol, raffinose and trehalose accumulation in cold-treated plants [12, 23, 63–67]. In this study, we detected an increased concentration of soluble sugar, trehalose, raffinose and galactinol in rice plants subjected to cold stress, and that key osmolyte synthesis enzymes were also significantly upregulated (Fig. 7). These findings suggest that these compounds play an important role in osmotic adjustment in rice during cold exposure.

## Conclusion

In this study, we identified 9317 differentially expressed genes in rice seedlings exposed to low temperature using high-throughput RNA-seq. An integrated analysis of physiological data and specific pathways indicated that several genes encoding enzymes involved in osmolyte synthesis were upregulated in rice plants exposed to cold stress and that these changes are correlated with increased levels of soluble sugars, trehalose, raffinose and galactinol in rice. High levels of carotenoid biosynthesis- and ABA biosynthesis-related transcripts correlate with carotenoid and ABA accumulation, respectively. However, chlorophyll content and Fv/Fm values decreased. This study has improved our understanding of the transcriptional responses of rice seedlings to low temperatures, which may help researchers increase rice productivity.

## Supplementary Information

**Additional file 1: Table S1.** Summary of RNA-Seq reads and their mapping on the rice genome.

**Additional file 2: Table S2.** Primers and corresponding sequences used for quantitative RT-PCR analysis.

**Additional file 3: Table S3.** Significantly-regulated DEGs at all the time points of chilling stress

**Additional file 4: Table S4.** DEGs associated with transcription factor families in rice at all five time points.

**Additional file 5: Table S5.** GO analysis of the 9317 DEGs under cold (2–48 h) conditions and 24 h recovery stages in Yu17S seedlings. (XLS 127 kb)

**Additional file 6: Table S6.** GO analysis of all the upregulated DEGs under cold (2–48 h) conditions in Yu17S seedlings.

**Additional file 7: Table S7.** GO analysis of all the upregulated and downregulated DEGs under 24 h recovery stages in Yu17S seedlings.

**Additional file 8: Table S8.** KEGG Pathway analysis of the 9317 DEGs under cold (2–48 h) conditions and 24 h recovery stages in Yu17S seedlings.

**Additional file 9: Table S9.** DEG associated with plant photosynthesis process in Yu17S seedlings in response to cold stress.

**Additional file 10: Table S10.** DEG associated with plant carotenoid biosynthesis in Yu17S seedlings in response to cold stress.

**Additional file 11: Table S11.** DEGs associated with phytohormone metabolism in Yu17S seedlings in response to cold stress.

**Additional file 12: Table S12.** DEG associated with osmotic adjustment in Yu17S seedlings in response to cold stress.

## Data Availability

Extra data has been appended as supplementary Tables. The accession number for sequence data generated in this study is PRJNA730675 available at http://trace.ncbi.nlm.nih.gov/.
