# Supplementary material for: Transcriptional and physiological data revealed cold tolerance in a photo-thermo sensitive genic male sterile line Yu17S
Source: BMC Plant Biol. 2022 Jan 21;22:44. doi: 10.1186/s12870-022-03437-8 (PMC8781465; doi:10.1186/s12870-022-03437-8)
Supplement: Supplementary file 1 — Additional file 1: Table S1. Summary of RNA-Seq reads and their mapping on the rice genome. [file 12870_2022_3437_MOESM1_ESM.docx]

**Table S1.** Summary of RNA-Seq reads and their mapping on the rice genome

| **Cold treatment** | | **Yu17s (cold tolerent variety)** | | | | | | | | | |
| --- | --- | --- | --- | --- | --- | --- | --- | --- | --- | --- | --- |
| **Hours of cold treatment** | **Temperature (^o^C)** | **Treatments** | | **Total reads** | **clean_reads** | **clean_bases** | **Q20** | **Q30** | **GC content**  **(%)** | **Total_map**  **(%)** | **Unique_map (%)** |
| 0 | 26 | T0 | 1 | 53619630 | 55016146 | 8.04G | 97.31 | 92.98 | 50.65 | 91.86% | 90.26% |
|  |  |  | 2 | 45847002 | 49826714 | 6.88G | 98.56 | 95.78 | 51.84 | 93.4% | 91.90% |
|  |  |  | 3 | 54466226 | 59530216 | 8.17G | 98.56 | 95.76 | 51.28 | 93.15% | 91.62% |
| 2 | 4 | T1 | 1 | 48814422 | 50952030 | 7.32G | 97.00 | 92.11 | 50.97 | 89.39% | 88.10% |
|  |  |  | 2 | 49315358 | 52865830 | 7.40G | 98.52 | 95.64 | 50.92 | 93.64% | 92.00% |
|  |  |  | 3 | 45320488 | 50258618 | 6.80G | 98.33 | 95.37 | 50.32 | 91.92% | 90.31% |
| 6 | 4 | T2 | 1 | 51597496 | 53249320 | 7.74G | 97.30 | 92.97 | 52.12 | 92.18% | 90.51% |
|  |  |  | 2 | 42496526 | 45694102 | 6.37G | 98.60 | 95.85 | 52.98 | 94.23% | 92.64% |
|  |  |  | 3 | 44114130 | 47865894 | 6.62G | 98.55 | 95.76 | 51.65 | 93.63% | 91.89% |
| 12 | 4 | T3 | 1 | 45103742 | 46015514 | 6.77G | 97.63 | 93.55 | 53.04 | 93.21% | 91.75% |
|  |  |  | 2 | 48601416 | 51840456 | 7.29G | 98.62 | 95.83 | 51.90 | 94.23% | 92.63% |
|  |  |  | 3 | 46534610 | 50782750 | 6.98G | 98.44 | 95.39 | 51.82 | 93.7% | 91.73% |
| 24 | 4 | T4 | 1 | 45269274 | 46386788 | 6.79G | 97.26 | 92.83 | 52.42 | 91.98% | 88.62% |
|  |  |  | 2 | 46808342 | 49928964 | 7.02G | 98.58 | 95.73 | 52.67 | 94.78% | 93.18% |
|  |  |  | 3 | 47439362 | 51522908 | 7.12G | 98.47 | 95.59 | 52.01 | 93.08% | 90.61% |
| 48 | 4 | T5 | 1 | 49891628 | 51756714 | 7.48G | 97.25 | 92.60 | 52.50 | 90.23% | 87.20% |
|  |  |  | 2 | 41850892 | 44652278 | 6.28G | 98.63 | 95.88 | 52.35 | 94.68% | 93.08% |
|  |  |  | 3 | 48143040 | 52372150 | 7.22G | 98.63 | 95.87 | 52.82 | 94.19% | 91.86% |
| 72* | 26 | R | 1 | 55741388 | 57343016 | 8.36G | 97.22 | 92.38 | 52.87 | 92.19% | 90.63% |
|  |  |  | 2 | 51768640 | 56340244 | 7.77G | 98.42 | 95.52 | 50.93 | 90.08% | 88.55% |
|  |  |  | 3 | 44784072 | 48414868 | 6.72G | 98.47 | 95.60 | 51.64 | 92.48% | 89.76% |

* The plants subjected to 48h of cold stress were kept at 26**^o^**C for 24h recovery. 1, 2, 3 represented three biological replicates in Yu17s under treatment.
